# Effect of Surface Coating on Bond Strength between Etched Feldspar Ceramic and Resin-Based Luting Agents

**DOI:** 10.1155/2018/3039251

**Published:** 2018-07-24

**Authors:** Jader Sebben, Volni A. Canevese, Rodrigo Alessandretti, Gabriel K. R. Pereira, Rafael Sarkis-Onofre, Ataís Bacchi, Aloísio O. Spazzin

**Affiliations:** Department of Restorative Dentistry, Graduate Program in Dentistry, Meridional Faculty, IMED, 99070-220 Passo Fundo, Brazil

## Abstract

This study evaluated adhesive protocols (silane, silane and unfilled resin, and universal adhesive) of bond strength between feldspar ceramic and resin-based luting agents (RBLAs). Thirty ceramic disks were embedded into acrylic resin, polished, etched, and randomly divided into 6 groups: S-RC: silane (S) and light-cured resin cement (RC) (RelyX Veneer; 3M ESPE); SB-RC: S followed by bond (B) (Clearfil SE Bond, Kuraray) and RC; UA-RC: universal adhesive (UA) (Single Bond Universal; 3M ESPE) and RC; flowable composite resin (F) was used on groups S-F, SB-F, and UA-F, and luting agent cylinders were built. The response variables (n=20) were microshear bond strength (MPa), characteristic strength (*σ*_*0*_, MPa), and Weibull modulus (*m*). The RC groups presented similar bond strengths regardless of whether or not bond was used. The S-F group with only silane application showed the highest bond strength, while the universal adhesive showed the lowest bond strength. The reliability was only affected in the UA-RC group, which was lower than the S-F group. Silane application is fundamental since the universal adhesive only decreased the bond strength between the feldspar ceramic and the RBLAs. Overall, the use of unfilled resin did not positively influence bond strength.

## 1. Introduction

Minimally invasive restorative techniques are recommended to preserve remaining dental structures and reduce pulp damage during dental preparation [1–3]. Feldspar ceramics are used to fabricate anterior laminate ceramic veneers with high survival rates [4, 5]; however, adhesive cementation using resin-based luting agents is fundamental to guaranteeing the high clinical survival of the ceramic restorations [6, 7].

The necessity of acid etching and silane application to improve bond strength between luting agents and glass ceramics is well documented [8]; however, an important and controversial factor is the use of hydrophobic unfilled resin (bond) on the etched ceramic surface after silane application to improve the bond strength with the resin-based luting agents. Some studies have shown that applying adhesive improves the bond strength between glass ceramics and luting agents [9, 10]; however, the use of unfilled resin on etched feldspar ceramic requires further evaluation. In addition, unfilled resin presents a low elastic modulus, which could reduce the strength of the feldspar ceramic [11], since studies have evidenced higher feldspar ceramic strengthening when using resin-based luting agents with a higher elastic modulus [11–14].

More recently, a new family of adhesive systems, known as universal or multimode adhesives, has been introduced, which may be used either as etch-and-rinse or as self-etch adhesives. This versatile new adhesion philosophy advocates using the simplest option for each strategy, that is, one-step self-etch or two-step etch-and-rinse [15] using the same single bottle of adhesive solution, which is much more challenging to dental substrates of different natures (i.e., sound, carious, sclerotic dentin, and enamel) [16]. Additionally, the matrix is based on a combination of monomers of hydrophilic (hydroxyethyl methacrylate/HEMA), hydrophobic (decanediol dimethacrylate/D3MA), and intermediate (bis-GMA) nature. This property combination allows universal adhesives to bridge the gap between the hydrophilic tooth substrate and the hydrophobic resin restorative under various surface conditions. Moreover, some universal adhesives may contain silane in their formulation, potentially eliminating the silanization step when bonding to glass ceramics or resin composites [17].

Another issue is the use of flowable composite resin as a luting agent to lute laminate ceramic veneers; however, different resin-based luting agents have distinct physical properties that could impact the strengthening and bond strength of the feldspar ceramic [14]. Therefore, this study compared different adhesive protocols (silane, silane and unfilled resin, and universal adhesive) to increase the bond strength between feldspar ceramic and a light-cured resin cement or a flowable composite resin. The null hypothesis was that the adhesive strategies would present similar bond strengths between the feldspar ceramic and the resin-based luting agents.

## 2. Materials and Methods

**Figure 1:**
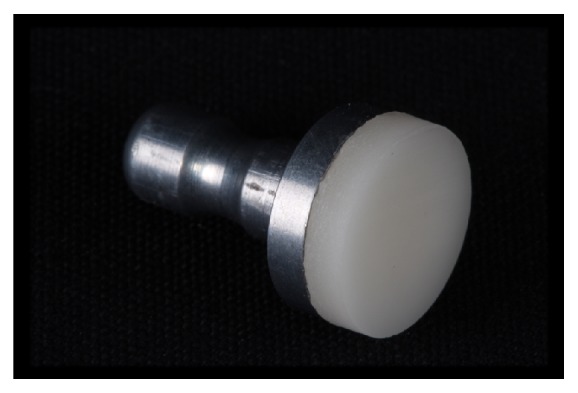
Feldspar ceramic block remnants (Vitablocs Mark II for Cerec; Vita Zahnfabrik) after polishing.

**Figure 2:**
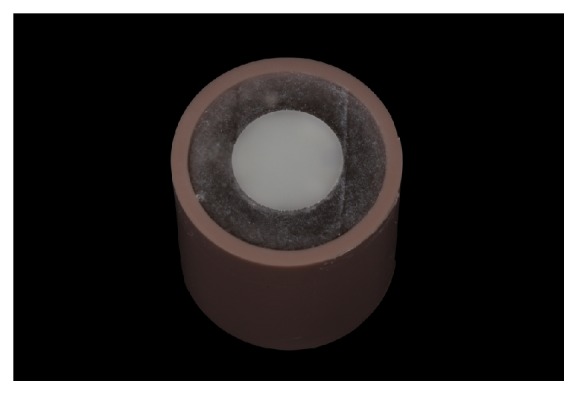
Specimen embedded into the PVC cylinder with acrylic resin.

CRIS (Checklist for Reporting In vitro Studies) guidelines adhered to this in vitro study [18]. Remnants of feldspar ceramic blocks (I14 A1C Vitablocs Mark II for Cerec; Vita Zahnfabrik) for CAD-CAM milling were sliced on a precision cutting machine (Isomet 1000; Buehler, Lake Bluff) and shaped manually by grinding with silicon carbide (SiC) paper (grit-sizes of #320, #400, #600, #800, and #1200) under water-cooling into disks of 12-mm diameter x 2-mm thickness (Figure 1). The ceramic disks were embedded into PVC cylinders with acrylic resin, and the top surface of the ceramic was again polished with the SiC paper (until the #1200 grit size) (Figure 2).

The embedded ceramic specimens were randomly divided into 6 groups based on adhesive protocol, as described in Table 1. The following adhesive procedures were performed by a calibrated operator. All specimens were submitted to the same surface treatment for enhanced adhesion to glass ceramics, that is, etching with 10% hydrofluoric acid for 90 seconds with posterior abundant water-rinsing for 30 seconds and air-drying for 30 seconds. Posteriorly, different adhesive protocols were investigated. On groups S-RC, SB-RC, S-F, and SB-F, a silane coupling agent (Ceramic Primer; 3M ESPE) was applied per the manufacturer's instructions; on groups SB-RC and SB-F, unfilled resin was applied (Clearfil SE Bond; Kuraray) in addition to the silane coupling agent; and on groups AU-RC and AU-F, only a universal one-step adhesive (Single Bond Universal; 3M ESPE) was applied (Table 1).

**Figure 3:**
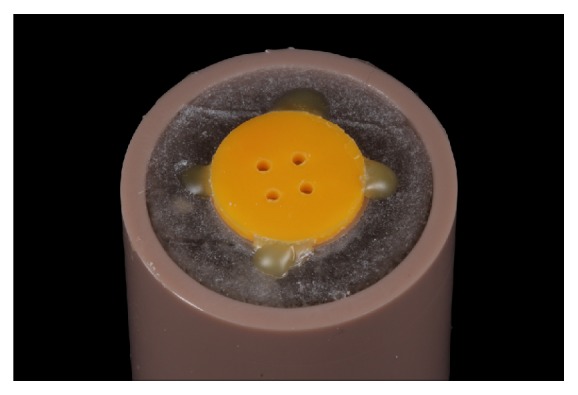
Elastomeric matrix (Oranwash L; Zhermack) fixed for obtaining the microshear samples.

**Figure 4:**
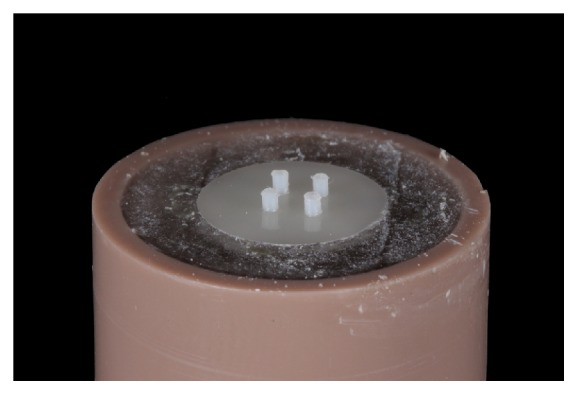
Four cylinders were obtained for the microshear bond strength test in each ceramic slice.

Finally, to obtain the samples for the microshear bond strength test, an elastomeric matrix (Oranwash L; Zhermack) with 4 equal cylinders of 1-mm diameter and 1-mm height was positioned over all ceramic specimen surfaces (Figure 3) to guide the cylinder manufacturing, which was executed by filling the matrix with resin cement (RelyX Veneer; 3M ESPE) or a flowable composite resin (Z350 Flow; 3M ESPE). When the cement/resin reached the top of the cylinder matrix, a polyester strip was positioned to remove any excess luting agent and regularize the top surface of the cylinder, flattened, and then light-cured for 60 seconds (Radii-Cal; SDI) (Figure 4).

One hundred twenty cylinders were manufactured, four per ceramic slice and 5 slices per adhesive protocol, thus yielding a sample size of 20 cylinders per group (n=20). For microshear bond strength testing, a universal testing machine was used (EMIC), in which a 0.2-mm-diameter steel wire was attached and carefully positioned around each cylinder (as close to the ceramic surface as possible, aligned to the bonding interface, and parallel to the attached loading cell). Next, an increasing load was applied at 1 mm/min until the cylinder finally detached from the ceramic surface. The data (maximum load to failure in Newtons [N]) was recorded, and the values of resin bond strength (MPa) for each cylinder were obtained using the following equation: stress = load/area. The failure modes of the tested samples were observed using an optical microscope (Model Stemi-2000C; Carl-Zeiss) at 40× magnification to classify the failure pattern as adhesive (between the ceramic and cement), cohesive (cohesive in the ceramic), or mixed.

All data passed normality and equal variance tests. Confidence intervals (95% CI) were calculated for bond strength. Groups were considered to significantly differ when the 95% confidence interval bounds did not overlap. A Weibull analysis was also performed on the bond strength data using statistical software (Minitab v.14; Minitab). The Weibull modulus (*m*), characteristic strength (*σ*_*0*_), and 95% upper and lower confidence limits were calculated using the maximum likelihood method.

## 3. Results

**Figure 5:**
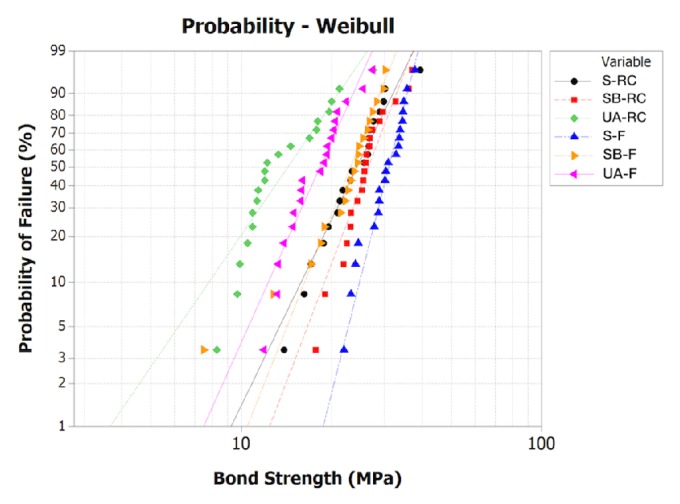
Weibull plot showing the failure probability (%) versus bond strength (MPa) for all experimental groups.

Table 2 presents the results for bond strength,* σ*_*0*_, and* m* for the tested groups. For the light-cured resin cement, the use of unfilled resin (SB-RC) or not (S-RC) presented similar bond strengths and* σ*_*0*_. For the flowable composite resin, the group with silane application alone (S-F) showed higher bond strength and* σ*_*0*_, followed by the group using the unfilled resin (SB-F). The lowest bond strength and* σ*_*0 *_were obtained when only the universal adhesive was used regardless of the resin-based luting agent (UA-RC and UA-F). The Weibull plot for all groups is shown in Figure 5. The bond interface reliability was only affected for the group in which the universal adhesive was used prior to the resin cement (UA-RC), which had lower* m* than the group using silane and flowable composite resin (S-F).

**Figure 6:**
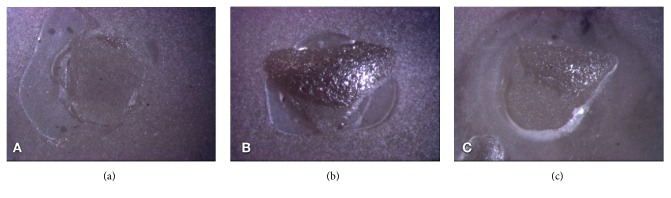
Representative images of the failure modes: (a) adhesive, (b) cohesive, and (c) mixed.

**Figure 7:**
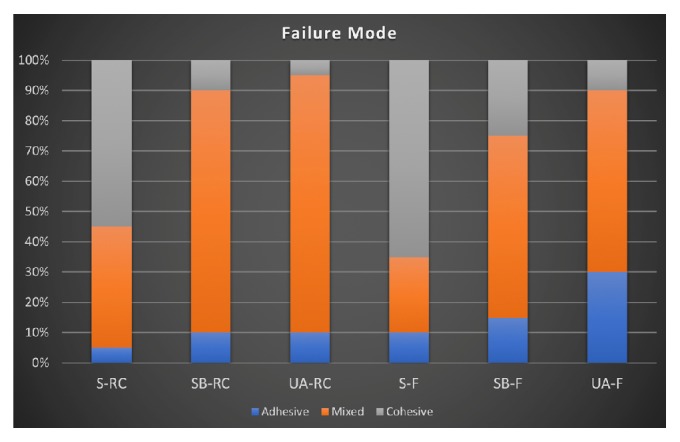
Failure modes for the experimental groups.

The failure mode is presented in Figures 6 and 7. The main failure type was cohesive (Figure 6(b)) for the groups where only silane was used prior to the luting agent. The other groups presented mainly mixed failures (Figure 6(c)). Overall, few adhesive failures were found, and the AU-F group presented the highest number (Figure 6(a)).

## 4. Discussion

The hypothesis tested in this study was rejected since different bond strength values were obtained depending on the type of resin-based luting agent and the adhesive strategy adopted. The use of a hydrophobic adhesive after silane application did not improve the bond strength between the feldspar ceramic and the resin-based luting agents in this evaluation. On the contrary, using this component provided lower bond strength values when associated with the flowable composite. The hypothesis that the adhesive layer could improve the bond strength has been suggested in previous studies [9, 10] in which the unfilled resin significantly increased the bond strength to leucite-reinforced and lithium disilicate-reinforced glass ceramic. The hypothesis for the increased bond strength was based on obtaining a deeper interpenetrated ceramic-resin layer due to the lower viscosity of the unfilled resin compared with the resin-based luting agents. This hypothesis, therefore, seems valid depending on the resin-based luting agent's viscosity, since the material used in the cited studies (Variolink II; Ivoclar Vivadent) had high filler content and consequently a high viscosity. Therefore, studies comparing the bonded interface using scanning electron microscopy could corroborate these suggestions.

In addition, the failure pattern analysis revealed higher cohesive failure rates for the groups where only the silane coupling agent was used and higher mixed failure rates for the groups with unfilled resin applied after silane. Therefore, a high bond strength near the cohesive strength of the feldspar ceramic could be suggested when only a silane coupling agent is used. For the materials adopted in the present study, the adhesive layer is not recommended since the interpenetrated layer is not likely increased, or, even worse, the additional layer formed by a material with a very low inorganic filler content could compromise the bond strength or decrease the feldspar ceramic's strength. This hypothesis, however, would be better elucidated by studying the interfaces.

Applying the universal adhesive provided significantly lower bond strength values irrespective of the luting agent used. The universal adhesive contains silane and 10-methacryloyloxy-decyl-dihydrogen-phosphate (10-MDP) as functional molecules to bond to the ceramic structure. Based on these results, the universal adhesives might not contain enough silane coupling agent in their formulations to ensure adequate chemical bonding to the ceramic, as their formulations are a combination of several components that differ from the silane agent available in specific bottles [19]. The 10-MDP is amphiphilic [19], meaning that vinyl and phosphate groups form hydrophobic and hydrophilic structures, respectively, to compose it. Therefore, the hydrophilicity of the universal adhesive might have impaired the bonding ability and stability of the interface compared with the other bonding strategies that were composed only of hydrophobic materials [19].

Results from a previous study were similar to those observed here. In that study, applying a silane coupling agent prior to the resin cement as a bonding strategy to a lithium disilicate-reinforced ceramic provided significantly higher bond strength (24.8 ± 3.1 MPa) than the universal adhesive (16.5 ± 2.4 MPa) [20]. Notably, the mean values in the former study are close to those of the present analysis, suggesting a reliable difference among these adhesive strategies.

The association of a silane coupling agent and a flowable composite resin provided higher bond strength obtained in the present study. The role of silane in glass ceramics is well-established since the alkoxy silane group of the coupling-agent covalently bonds with the ceramic structure [21]. More specifically, hydrolysable functional groups react with the surface hydroxyl groups of inorganic substrates, creating a siloxane bond (Si-O-Si) [22]. The organic nonhydrolysable functional group with a C=C double bond can polymerize with resin composite monomers containing double bonds [19–21, 23].

The higher bond strength found for the flowable composite resin group compared with the resin cement after silane application might be related to a higher filler content and filler composition that improved the material's strength. Per the manufacturer's information, the flowable composite resin contains silica nanoparticles (75 nm), zirconia fillers (5-10 nm), and clusters (0.6-1.4 *μ*m), whereas the resin cement is composed only of microparticles with an average size of 0.6 *μ*m. The specimens were not submitted to artificial aging, such as thermal or mechanical cycling, being the limitation of this study. In addition, further studies should be made evaluating the mechanical behavior of the luted feldspar ceramic in these conditions.

## 5. Conclusions

Silane application is fundamental to obtaining improved bond strength since using only a universal adhesive decreased the bond strength between the feldspar ceramic and the resin-based luting agents. Overall, the use of unfilled resin did not positively influence the bond strength.

## Figures and Tables

**Table 1:** Study design.

Groups	Silane coupling agent	Adhesive	Resin-based luting agents
S-RC	Ceramic Primer (3M ESPE)	-	RelyX Veneer (3M ESPE)
SB-RC		Clearfil SE Bond (Kuraray), air-dried for 5 seconds	
UA-RC	-	Single Bond Universal (3M ESPE), kept for 15 seconds and air-dried for 5 seconds	

S-F	Ceramic Primer (3M ESPE)	-	Z350 Flow; 3M ESPE
SB-F		Clearfil SE Bond (Kuraray), air-dried for 5 seconds	
UA-F	-	Single Bond Universal (3M ESPE), kept for 15 seconds and air-dried for 5 seconds	

**Table 2:** Estimates (95% confidence intervals) for mean microshear bond strength (*μSBS*), characteristic strength (*σ*_*0*_), and Weibull modulus (*m*).

Group	*μSBS* (MPa)	*σ*_*0*_ (MPa)	*m*
S-RC	24.2 (21.8–26.7)^B^	26.5 (23.8–29.4)^B^	4.4 (3.2–6.0)^AB^
SB-RC	26.3 (24.2–28.5)^AB^	28.3 (26.0–30.8)^AB^	5.6 (4.1–7.7)^AB^
UA-RC	14.4 (12.3–16.6)^C^	16.1 (13.8–18.7)^C^	3.1 (2.3–4.3)^B^
S-F	30.5 (28.5–32.5)^A^	32.4 (30.6–34.2)^A^	8.4 (5.9–12.0)^A^
SB-F	22.8 (20.4–25.3)^B^	24.7 (22.7–27.0)^B^	5.3 (3.7–7.7)^AB^
UA-F	18.2 (16.4–20.0)^C^	19.8 (17.9–21.8)^C^	4.7 (3.4–6.6)^AB^

Distinct letters in the same column indicate significant differences between groups.

## Data Availability

The data used to support the findings of this study are available from the corresponding author upon request.
